# Pharmacological activation of TRPV4 produces immediate cell damage and induction of apoptosis in human melanoma cells and HaCaT keratinocytes

**DOI:** 10.1371/journal.pone.0190307

**Published:** 2018-01-02

**Authors:** Aida Olivan-Viguera, Angel Luis Garcia-Otin, Javier Lozano-Gerona, Edgar Abarca-Lachen, Ana J. Garcia-Malinis, Kirk L. Hamilton, Yolanda Gilaberte, Esther Pueyo, Ralf Köhler

**Affiliations:** 1 Biosignal Interpretation and Computational Simulation (BSICoS), Aragón Institute of Engineering Research (I3A), University of Zaragoza, Zaragoza, Spain; 2 Instituto de Investigación Sanitaria (IIS) Aragón, Zaragoza, Spain; 3 Aragón Institute of Health Sciences (IACS), Zaragoza, Spain; 4 Universidad San Jorge, Faculty of Health Sciences, Villanueva de Gállego, Spain; 5 Dept. of Dermatology, Hospital San Jorge, Huesca, Spain; 6 Dept. of Physiology, School of Biomedical Sciences, University of Otago, Dunedin, New Zealand; 7 Dept. of Dermatology, University Hospital Miguel Servet, Zaragoza, Spain; 8 Biomedical Research Networking Center in Bioengineering, Biomaterials and Nanomedicine (CIBER-BBN), 50018-Zaragoza, Spain; 9 Aragón Agency for Research and Development (ARAID), Zaragoza, Spain; University of Alabama at Birmingham, UNITED STATES

## Abstract

**Background:**

TRPV4 channels are calcium-permeable cation channels that are activated by several physicochemical stimuli. Accordingly, TRPV4 channels have been implicated in the regulation of osmosensing, mechanotransduction, thermosensation, and epithelial/endothelial barrier functions. Whether TRPV4 is also mechanistically implicated in melanoma cell proliferation is not clear. Here, we hypothesized that TRPV4 is expressed in human melanoma and that pharmacological activation interferes with cell proliferation.

**Methodology/Principal findings:**

TRPV4 functions were studied in melanoma cell lines (A375, SK-MEL-28, MKTBR), immortalized non-cancer keratinocytes (HaCaT), and murine 3T3 fibroblasts by patch-clamp, qRT-PCR, intracellular calcium measurements, cell proliferation, and flow cytometric assays of apoptosis and cell cycle. The selective TRPV4-activator, GSK1016790A, elicited non-selective cation currents with TRPV4-typical current-voltage-relationship in all cell lines. GSK1016790A-induced currents were blocked by the TRPV4-blocker, HC067047. TRPV4 mRNA expression was demonstrated by qRT-PCR. In A375 cells, TRPV4 activation was frequently paralleled by co-activation of calcium/calmodulin-regulated KCa3.1 channels. Light microscopy showed that TRPV4-activation produced rapid cellular disarrangement, nuclear densification, and detachment of a large fraction of all melanoma cell lines and HaCaT cells. TRPV4-activation induced apoptosis and drastically inhibited A375 and HaCaT proliferation that could be partially prevented by HC067047.

**Conclusions/Significance:**

Our study showed that TRPV4 channels were functionally expressed in human melanoma cell lines and in human keratinocytes. Pharmacological TRPV4 activation in human melanoma cells and keratinocytes caused severe cellular disarrangement, necrosis and apoptosis. Pharmacological targeting of TRPV4 could be an alternative or adjuvant therapeutic strategy to treat melanoma progression and other proliferative skin disorders.

## Introduction

The transient receptor potential channel subtype 4, TRPV4, is a poly-modally regulated channel with a considerable Ca^2+^-permeability that is capable of transducing a broad variety of physicochemical stimuli into intracellular calcium signals (for extensive review and key papers see [1,2,3,4,5,6,7]. In biology, the channel has therefore been suggested to serve as physiological osmo/mechanosensor, thermosensor, in epithelia/endothelia in several tissues, such as e.g. arteries, lungs, kidneys, and skin (for extensive reviews see [2,8]). Moreover, pharmaceutical companies have considered TRPV4 a promising drug target to treat disease, e.g. bladder dysfunction [9], sepsis [10], and pulmonary edema [11,12], giving rise to novel selective small molecule modulators, such as the activator, GSK1016790A [13], and several selective inhibitors [1]. In the following, experimental proof-of-concept studies revealed that inhibition of TRPV4 was capable of preventing and resolving pulmonary edema caused by heart failure [11], while the TRPV4-activator, GSK1016790A, produces endothelial/epithelial barrier disruption, lung edema, and circulatory collapse in rodents [12,14]. In humans, mutations in the TRPV4 gene—supposedly gain-of-function mutations that may result in cytotoxic calcium-overload—caused skeletal dysplasia and motor and sensory neuropathies of very variable severity (for in-depth review see [8]). While the precise physiological or pathophysiological role of TRPV4 and the precise molecular mechanism regulating activity of the channel remain not well defined yet, the existing insight suggested substantial cytotoxicity of TRPV4 over-activation.

Today there is not much known about roles of TRPV4 in neo-proliferative disease. So far, TRPV4 in breast cancer cells has been shown to promote cancer cell extravasation and to be a marker of poor prognosis of several solid epithelial cancers [15]. In addition, endothelial TRPV4 has been shown to regulate tumor angiogenesis [16]. However, whether TRPV4 is directly involved in the regulation of cancer cell proliferation, is unknown.

Melanoma is a skin cancer with high incidence and mortality and it is caused by oncogenic transformation of melanocytes that are located in the deeper layers of the epidermis and produce melanin that acts as light absorber, biologically meant to provide photo protection against UV-radiation of sunlight and subjected to complex local and neuroendocrine regulation (for in-depth review see [17,18,19,20].

In the present study, we tested the hypothesis that 1) TRPV4 is functionally expressed 5 and that 2) pharmacological manipulation of TRPV4 interferes with melanoma cell proliferation.

In the following, we show that human melanoma cell lines expressed functional TRPV4 channels and that the TRPV4-activator, GSK1016790A, caused a strong calcium-overload and cellular disarrangement, increased the rate of apoptosis, and strongly inhibited cell proliferation/survival. Similarly, GSK1016790A induced apoptosis and impeded proliferation of HaCaT keratinocytes, a spontaneously immortalized aneuploid keratinocyte cell line from human skin. Together, this suggested that pharmacological TRPV4 activators could have utility as inhibitors of melanoma growth and disease progression and possibly other proliferative skin conditions.

## Methods

### Cell lines

The amelanotic A375, melanotic SK-MEL-28 and uveal MKTBR melanoma cell lines, HaCaT keratinocytes (all gifts from by Dr. Martin-Duque, Aragón Institute of Health Sciences, Zaragoza), and 3T3 murine fibroblasts (gift of Dr. Arbonés-Mainar, Aragón Institute of Health Sciences, Zaragoza) were cultured in DMEM supplemented with 10% newborn calf serum (NCS) and streptomycin and penicillin (S/P, all from Biochrom KG, Berlin, Germany). HaCaT cells were grown in GBMI medium supplemented with 10% NCS and S/P. Melanoma cell lines were used at passage 17 and at passage 4 for HaCaT. For proliferation and apoptosis assays, NCS concentration was 1%. For patch-clamp experiments cells were seeded on coverslips and investigated within 24 h.

### Patch-clamp electrophysiology

Membrane currents were measured in the whole-cell configuration using an EPC10-USB amplifier (HEKA, Electronics, Lambrecht-Pfalz, Germany), a clamp protocol consisting of voltage pulses (-80,+80, 0 mV, for 200 ms each) and voltage-ramps (-100 to +100, 500 ms), borosilicate patch-pipettes with a tip resistance of 4–6 MΩ, a high K^+^ pipette solution containing 200 nM “free”Ca^2+^ and a high Na^+^ bath solution, as described in more detail previously [21]. For data acquisition and analysis, we used the patch-master program (HEKA). Ohmic leak currents of up to 1 nS were subtracted where appropriate.

### RNA isolation and qRT-PCR studies

RNA from cultured passage 4 HaCaT and passage 17 A375 cells was isolated with TriReagent (Sigma, Saint-Louis, MO) following the manufacturer’s protocol, and further purified using RNA Clean-up and concentration Micro-Elute kit (Norgen Biotek, Thorold, Canada). In another series of experiments, RNA was isolated from frozen aliquots of A373, MKRBR, and SK-MEL-28. Genomic DNA elimination was accomplished by using Ambion DNA free kit (Invitrogen, Carlsbad, CA). Quantity and purity of extracted RNA samples were analyzed by spectrophotometry (NanoDrop 1000, Thermofisher, Waltham, MA) before using them to reverse transcription or storing them at –80°C for later use. Isolated RNA samples were analyzed for integrity and genomic DNA contamination by gel electrophoresis prior to their use in reverse transcription. For TRPV4 and KCa3.1, reverse transcription was performed using 600 ng and 1500 ng of total RNA, respectively, in a final volume of 20 μl by using the SuperScript IV reverse transcriptase and random hexamers (both from Invitrogen, Carlsbad, CA) following the manufacturer’s protocol.

For qPCR, we used 2 μl (TRPV4) and 0.3 μl (KCa3.1) of transcription products, and corresponding amounts for detection of GAPDH as reference gene, and employed the SYBR Select Master Mix (Applied Biosystems, Foster City, CA) according to the manufacturer’s protocol. Primers were: KCa3.1-F, 5’-CATCACATTCCTGACCATCG-3’, KCa3.1-R, 5’-ACGTGCTTCTCTGCCTTGTT-3’, TRPV4-F, 5’-GATCTTTCAGCACATCATCC-3’, TRPV4-R, 5’-GGTCATAAAGCGAGGAATAC-3’, GAPDH-F, 5’-GGGATCAATGACCCCTTCAT-3’, GAPDH-R, 5’-GCCATGGAATTTGCCAT-3’. Amplifications were carried out in triplicates using a Step One Plus thermocycler (Applied Biosystems, Foster City, CA) and the following cycle program: 95° C, 15 sec and 60° C, 60 sec repeated for 40 cycles. Thereafter, a melting curve was run to verify amplification products. Collected data were analyzed with LinRegPCR software [22] and derived Cq values and mean efficiencies of reaction were used to calculate expression of the gene of interest (GOI) relative to GAPDH using the formula: % *of GAPDH* = *Efficiency*^*Cq*(*GAPDH*)–*Cq*(*GOI*)^*x*100.

### Optical mapping of intracellular calcium

A375 or HACAT cells were plated on coverslips and cultured until 90% confluence. Coverslips were immersed into Tyrode´s solution (in mM, 140 NaCl, 4.5 KCl, 1 MgCl_2_, 1.8 CaCl_2_, 10 Glucose, 10 HEPES, pH 7.4) and cells were incubated at 37°C with Rhod-2 AM (Invitrogen, Carlsbad, CA) plus Pluronic F127 (20% solution in DMSO, Invitrogen, Carlsbad, CA) at 5 μM for 40 min. Coverslips were washed twice in Tyrode´s solution, and incubated for another 30 min to allow Rhod-2 AM de-esterification. Measurements were conducted within ≈4 hours at room temperature. Recordings were conducted over 4 min at a sampling rate of 10 frames per sec. Compounds or vehicle (DMSO, 0.1%) were slowly washed into the bath solution. Fluorescence was recorded with a high speed and low noise optical mapping device consisting of a MiCAM O5-Ultima CMOS camera (SciMedia, Costa Mesa, CA) with a spatial resolution of 240x256 pixels, 30x30 μm per pixel, temporal resolution of 1 ms, and a 150 W halogen lamp (HL-151, SciMedia) with a built-in electromagnetic shutter to minimize photo bleaching. Excitation was done at 530 nm. Emitted fluorescence was split with a dichroic mirror (560 nm) and detected with camera equipped with a 575/15 nm band pass filter for Rhod-2 AM fluorescence recordings. Fluorescence recordings were analyzed with the BV Ana imaging software (SciMedia). Four regions of Interest (ROI) were selected, corresponding to four clusters of approx. 30–80 pixels. Average intensity of fluorescence was analyzed for each ROI and data points were smoothed with a 3x3 pixels spatial median filter. Background fluorescence was subtracted automatically.

### Annexin V/PI double-staining assay

Apoptotic cells were quantified by flow cytometry using an Annexin V-FITC detection kit (Immunostep, Salamanca, Spain). A375 cells were seeded in 6-well plates and grown to 60–70% confluence. Then cells were treated with DMSO (vehicle) at 0.2%, GSK1016790A (10 nM), or with GSK1016790A and HC067047 (1 μM) for 1 h, 24 h, or 72 h at 37°C. Thereafter adherent cells were gently detached by using Accutase (Corning Inc, Corning, NY) and cells were stained with Annexin V-FITC and propidium iodide (PI) according to the manufacturer’s instructions. Cells were measured in a FACSAria flow cytometer (BD Bioscience, Madrid, Spain) and data were analyzed with the Kaluza Flow Cytometry Analysis Software v1.1 (Beckman Coulter, Brea, CA).

### Cell viability analysis with trypan blue staining

Cells spontaneously detached after exposition to DMSO (0.2%), GSK1016790A (10 nM) or GSK1016790A plus HC067047 (1 μM) during 1 h, 24 h or 72 h were collected from cell culture media and stained with trypan blue (Invitrogen, Carlsbad, CA) to assess viability. Counting was performed in a Neubauer hemocytometer following standard procedures.

### Cell cycle analysis

Cell cycle in A375 cells was analyzed by flow cytometry by assessing cell DNA content. Cells were harvested after gentle Accutase treatment and centrifuged at 250xg during 5 min. The cell pellet was re-suspended in D-PBS (Lonza, Portsmouth, NH) before fixation with ice-cold ethanol under agitation. Fixed cells were stored at 4°C for 24 h, then treated with RNAse (Sigma-Aldrich, Tres Cantos, Madrid, Spain), stained with PI (Sigma-Aldrich), and analyzed in a Gallios flow cytometer (Beckman Coulter, Brea, CA). Data were analyzed with Modfit LT v3.3 software (Verity Software House, Topsham, ME).

### Giemsa stain

Cells were fixed with 100% methanol and dried. Thereafter, cells were stained with a ready-to-use Giemsa solution (Sigma-Aldrich) for 30 min followed by extensive washing with deionized water. Photographs of cells were taken with a digital camera.

To quantify cell detachment, cells in 1 ml supernatant were treated with trypan blue and viable and non-viable cells were counted with a Neubauer chamber.

### Cell proliferation assays

Cell proliferation/survival was spectrophotometrically assessed using the Janus Green B green assay as described previously with some modifications [23]. Briefly, cells (1500 cells/well) were seeded in 96-well plates and the compound(s) or the vehicle, DMSO, were added. Vehicle concentrations were kept the same for all concentrations of compounds and their combinations. Cells were formalin-fixed at day 0 (immediately after addition of compounds),1,2, 3, and 4. Fixed cells were stained for 5 min with 50 μl/well of 0.3% Janus B Green dye (Acros Organics, Belgium) at room temperature with continuous stirring followed by a washing step with water. The dye was eluted with 200 μl/well of 0.5 M HCl of hydrochloric acid and top-read measurements of absorbance were performed in a microplate reader (Sinergy HT, Biotek, USA) at 595 nm. Data in Figures are presented as % of control (DMSO) for clarity. For comparisons absorption values were used.

### Compounds

GSK1016790A (N-[(2S)-1-(4-(N-[(2,4-Dichlorophenyl)sulfonyl]-L-seryl)-1-piperazinyl)-4-methyl-1-oxo-2-pentanyl]-1-benzothiophene-2-carboxamide) and HC067047 (2-Methyl-1-[3-(4-morpholinyl)propyl]-5-phenyl-N-[3-(trifluoromethyl)phenyl]-1H-pyrrole-3-carboxamide) were purchased from Tocris Bioscience (Bristol, UK) and 1 mM or 10 mM stock solutions, respectively, were prepared with DMSO and stored at -20 C. At the day of experimentation, stock solutions were further stepwise pre-diluted with first DMSO giving concentrations of 0.1 μM-1 mM for GSK1016790A and then 1:10 with the bath solution or culture media. Appropriate amounts were added to wells or the bath chamber giving final concentrations of 0.01 nM– 10 μM. The final concentration of DMSO was 0.1% - 0.2%). RA-2 (1,3-Phenylenebis(methylene) bis(3-fluoro-4-hydroxybenzoate) was synthesized as described previously [21]. SKA-121 (5-Methylnaphtho[2,1-d][1,3]oxazol-2(3H)-imine) was a kind gift from Prof. Heike Wulff, Pharmacology at University of California, Davis. 13b ([3,5-Bis[(3-fluoro-4-hydroxy-benzoyl)oxymethyl]phenyl]methyl 3-fluoro-4-hydroxy-benzoate) was a kind gift from Prof. Robert Kiss, Laboratoire de Toxicologie, Institut de Pharmacie, ULB, Belgium. All other drugs were purchased from Sigma (Deisenhofen, Germany).

### Statistics

Data are given as means ± SEM. Data sets were compared with the paired or unpaired Student’s T test or ANOVA, where appropriate. P-values of 0.05 were considered statistically significant.

## Results

### Electrophysiological identification of TRPV4 in melanoma cell lines

**Fig 1:**
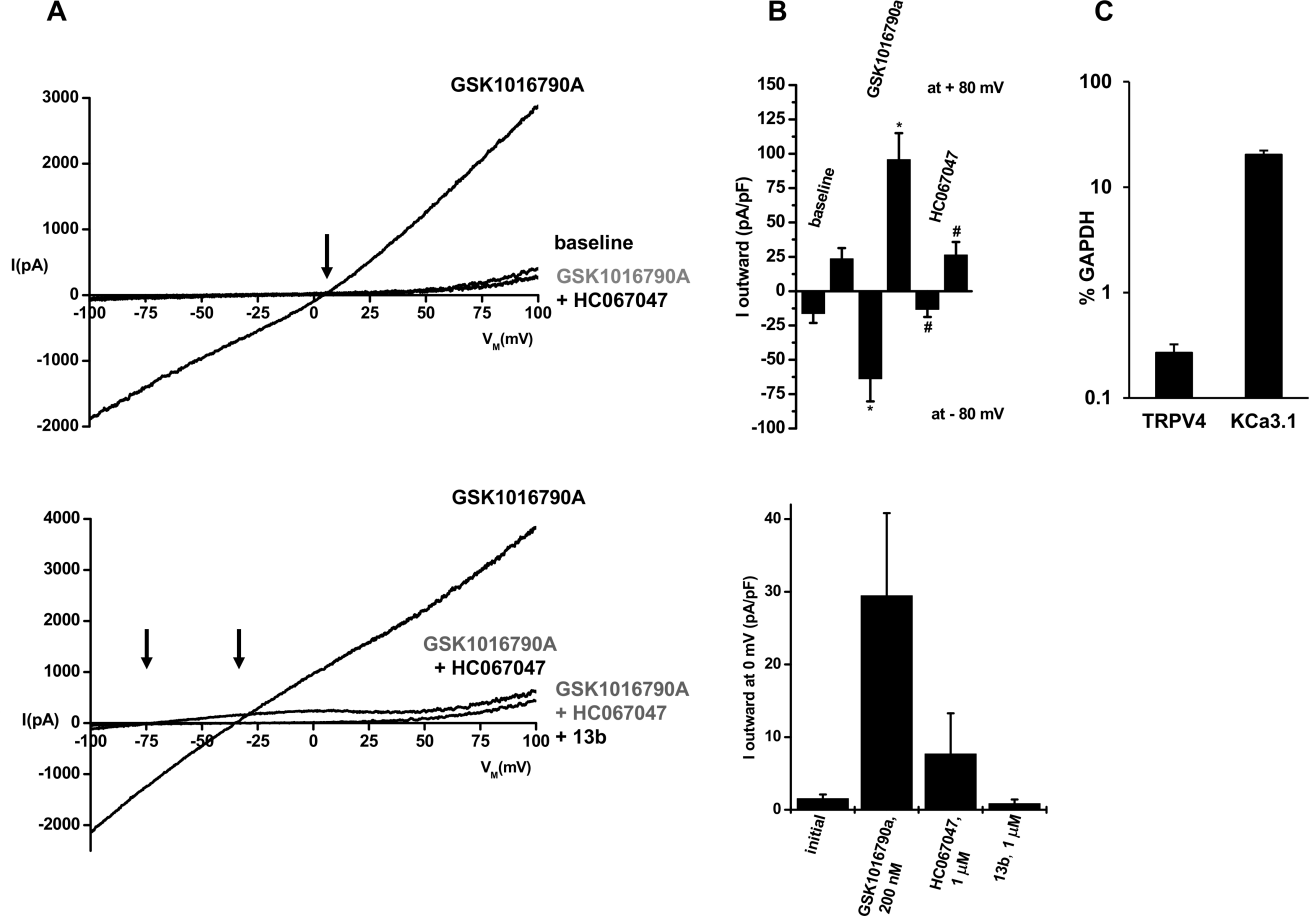
Characterization of TRPV4 channels in A375 melanoma cells. A) Upper panel: Exemplary whole-cell recordings showing activation of TRPV4 channels by GSK1016790A (200 nM) and inhibition of currents by HC067047 (1 μM). The arrow indicates a positive reversal potential of GSK1016790A-activated currents. Baseline currents were not considerable inhibited by HC067047. Lower panel: Co-activation of K_Ca_-currents. The right arrow indicates a negative reversal potential (E_rev_) of ca. -35 mV of the mixed TRPV4 and K_Ca_ current and the left arrow indicates an E_rev_ of ca. -75 mV of the isolated K_Ca_-current after inhibition of TRPV4 currents by HC067047. The K_Ca_-current was fully blocked by the negative-gating modulator of KCa3.1 channels, 13b (1 μM). B) Upper panel: Mean normalized currents at clamp potentials of -80 and +80 mV before and after addition GSK1016790A (n = 8, experiments) and after addition of HC067047 (n = 8). Lower panel: Mean mixed TRPV4/KCa3.1 currents at a clamp potential of 0 mV after addition of GSK1016790A (n = 5) and inhibition of TRPV4 currents by HC067047 (n = 4) and of KCa3.1 currents by 13b (n = 5). C) Quantitative RT-PCR analysis of TRPV4 and KCa3.1 gene expression as percentage of GAPDH expression (replicates, n = 3). Data points are means ± SEM.

In whole cell patch-clamp experiments in A375 melanoma cells (Fig 1A and 1B), the TRPV4-opener, GSK1016790A instantaneously evoked large cation currents that reversed at slightly positive potentials (E_rev_, ≈+4 mV, Fig 1A, upper panel). The IV relationship showed mild outward-rectification with larger currents at +80 mV than at -80 mV and was slightly N-shaped with a lower slope at potentials ranging from -50 mV to 0 mV. The selective blocker of TRPV4, HC067047 [11], abolished this current, but it took 3–4 min to achieve full channel blockade at 1 μM. Together, these biophysical and pharmacological footprints are characteristic for TRPV4 [1,4].

In 5 out 8 experiments, TRPV4 activation was accompanied by activation of a K^+^ current causing a shift of E_rev_ to more negative values (≈-35 mV; Fig 1A and 1B, lower panels). Inhibition of TRPV4 with HC067047 unmasked this K^+^ current, which then showed strong inward-rectification and reversed at ≈-75 mV, which is near the K^+^ equilibrium potential. The negative-gating modulator of small/intermediate-conductance KCa2/3 channels, 13b (1 μM, Fig 1A and 1B, lower panels)[24] fully blocked this K^+^ current. These properties identified this co-activated K^+^ channel as the calcium/calmodulin-regulated KCa3.1 channel [25,26,27].

In Fig 1, we demonstrated functional expression of TRPV4 and KCa3.1 in the A375 melanoma cells and we additionally determined mRNA expression of TRPV4 and KCa3.1 in these cells by qRT-PCR (Fig 1C).

**Fig 2:**
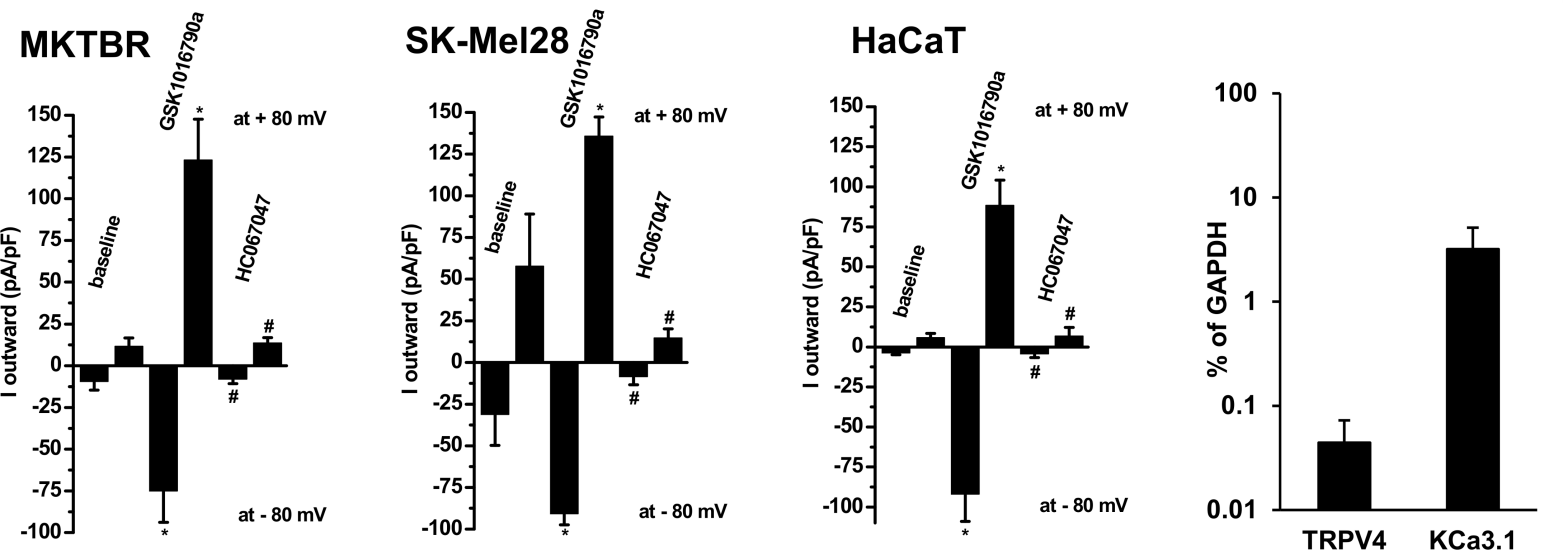
GSK1016790A-induced TRPV4-currents and inhibition by HC067047 in the melanoma lines, MKTBR and SK-MEL-28, and the human non-cancer keratinocyte line, HaCaT. Data points are means ± SEM (cells, n = 4–6 each). Panel on right: Quantitative RT-PCR analysis of TRPV4 and KCa3.1 gene expression in HaCaT as percentage of GAPDH expression (replicates, n = 3). Data points are means ± SEM.

Concerning the other two melanotic melanoma lines, MKTBR and SK-MEL-28, we measured similar TRPV4 currents and found similar expression levels for TRPV4 channels while KCa3.1 expression appeared to lower to some degree in MKTBR and SK-MEL-2 if compared to A375 cells (Fig 2 and S1 Fig).

In the non-cancer human keratinocyte line HaCaT, TRPV4 currents were also found, but inward-rectification was less evident (see summary data in the bar chart in Fig 2), suggesting co-activation of other perhaps calcium-activated non-selective cation channels with a linear current-voltage relationship or Cl^-^ channels, which, however, were not further characterized here. We did not observe co-activation of KCa3.1 currents. We found mRNA transcripts for both channels and expression levels were approx. 10-times lower than in A375 (Fig 2, on right).

Together, we proved functional TRPV4 expression in melanoma cells and immortalized human keratinocytes.

In 3T3 fibroblasts we also found activation of TRPV4 by GSK1016790A and sensitivity of the current to HC067047. But current amplitudes were considerably smaller than those in the other cell lines (S3 Fig). Moreover, mRNA expression levels might be low because we did not see amplification with our 40-cycles qRT-PCR protocol (data not shown).

### TRPV4-mediated changes in intracellular calcium

**Fig 3:**
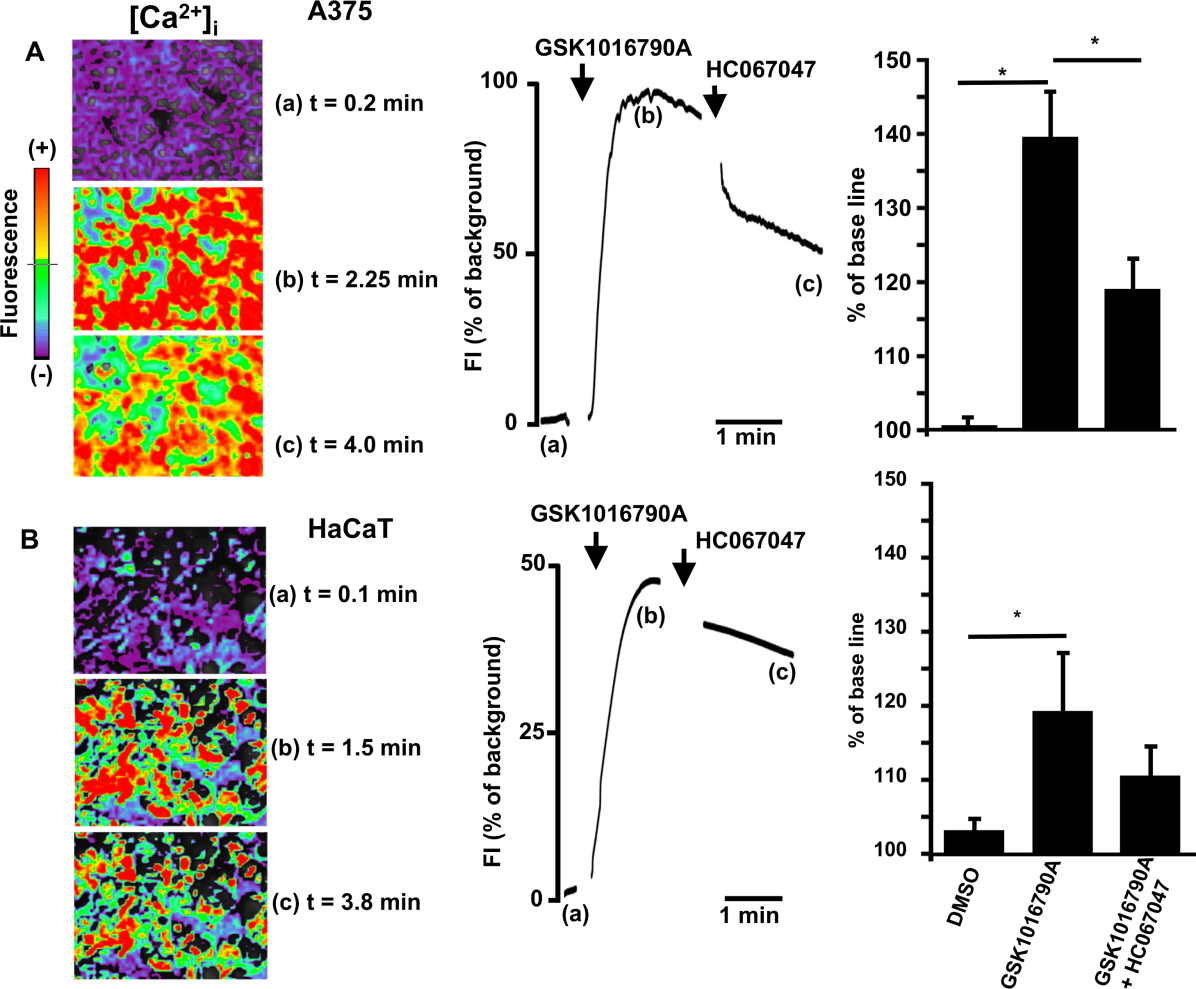
A) Changes of intracellular calcium ([Ca^2+^_i_]) in response to TRPV4 activation in A375 cells. Exemplary images of GSK1016790A(1 μM)-induced increase of [Ca^2+^_i_] (upper left panel), its time course (upper panel in middle), and (on right) summary data of % change of fluorescence to GSK1016790A (n = 10 independent experiments) and in combination with HC067047 (n = 5 independent experiments). HC067047 (10 μM) partially reversed the response. Note that artifacts caused by compound addition were removed. No change of fluorescence was recorded in the presence of vehicle (DMSO 0.1%, n = 3). B) Lower panel on left: Exemplary images of changes of intracellular calcium ([Ca^2+^]_i_) to TRPV4 activation in HaCaT cells. Note that artefacts caused by compound addition to the bath were removed. In middle: Time course of changes in [Ca^2+^]_i_ in response to GSK1016790A (n = 4 independent experiments) alone and in combination with HC067047 (n = 4 independent experiments). The vehicle (DMSO 0.1%, n = 3) produced virtually no response. On right: Summary of data. Data are means ± SEM; *P<0.001, Student’s T test.

Since TRPV4 is a Ca^2+^-permeable channel, we next studied TRPV4-mediated increases in [Ca^2+^]_i_ in A375 and HaCaT cells by optical mapping (Fig 3A and 3B). GSK1016790A produced a fast and long-lasting increase of intracellular calcium in A375 cells. HC067047 reversed -though incompletely- this response. Similar responses were seen in HaCaT cells, although the amplitude of the response was approx. half of that seen in A375 cells.

Hence, TRPV4 activation caused strong elevations in [Ca^2+^]_i_ in these cell lines.

### Morphological alteration caused by TRPV4 activation

**Fig 4:**
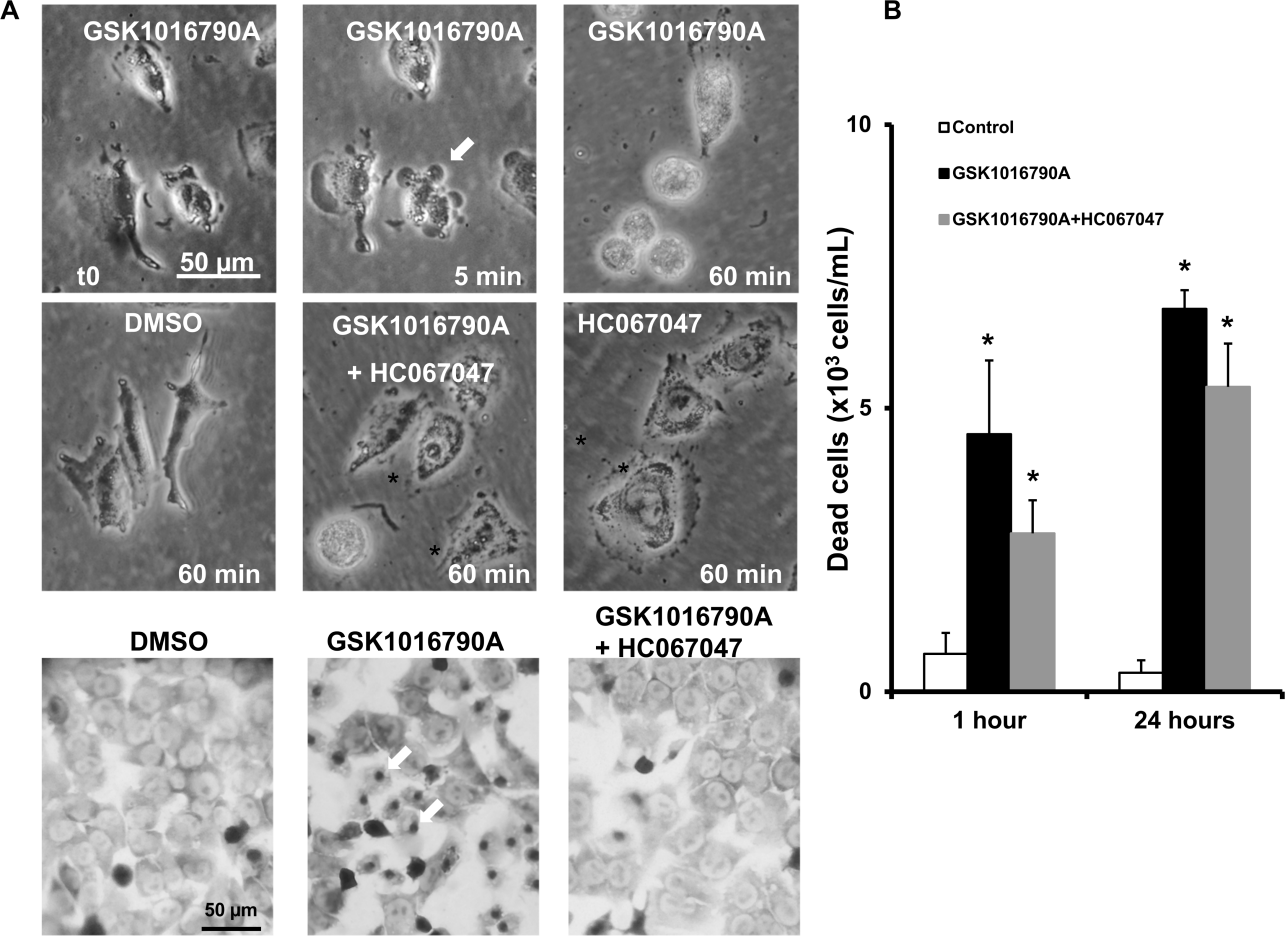
Alterations of cell morphology, cell detachment and cell death induced by GSK1016790A. A) Upper two panels: Exemplary light microscopic images illustrating time course of cell retraction, membrane blebbing (indicated by arrows), and cell detachment during the first hour of exposure to GSK1016790A (1 μM). HC067047 (1 μM) prevented visibly GSK1016790A-induced changes. HC067047 or vehicle (DMSO) had no visible effect. Lower panel: Giemsa-stained A375 cells after 1 h exposure to GSK1016790A, in combination with HC067047, or DMSO. Note the densification of nuclei (dark-grey dots indicated by arrows) in GSK1016790A-treated cells. B) Counts of non-viable, “death” cells in supernatant. Data points are means ± SEM (number of independent experiments, n = 3). *P<0.05 vs. DMSO, #P <0.05 vs. GSK1016790A; Student’s T test.

To determine if activation of TRPV4 has any detrimental effects on A375 melanoma cells, we next tested whether TRPV4-activation produced alterations in cell morphology and monitored alive A375 cells under a light microscope over 60 min. As shown in Fig 4A, challenging A375 cells with GSK1016790A produced overt retraction of lamellipodia, rupture of intercellular junctions, blebbing, and densification of cell nuclei (pyknosis), and cell swelling and detachment. Accordingly, we found a high number of necrotic (non-viable) cells in the supernatant over a 3-days period (Fig 4B). These immediate and longer-lasting alterations could be prevented by HC067047 to a considerable extent (Fig 4A and 4B). The vehicle, DMSO, or HC067047 did not affect cell morphology and cell detachment in a significant fashion. Such alterations were also seen in MKTBR cells (S2 Fig, upper panel on left). In HaCaT keratinocytes, GSK1016790A also produced blebbing (S2 Fig, upper panel on right), nuclear densification, and high numbers of necrotic (non-viable) cells in the supernatant (S2 Fig, lower panels. Such morphological alterations were not observed in 3T3 fibroblasts (S3 Fig), which may be explained by the substantially lower amounts of inducible currents that may not lead to excessive calcium and sodium entry.

### Flow cytomteric measurements of TRPV4-mediated apoptosis

**Fig 5:**
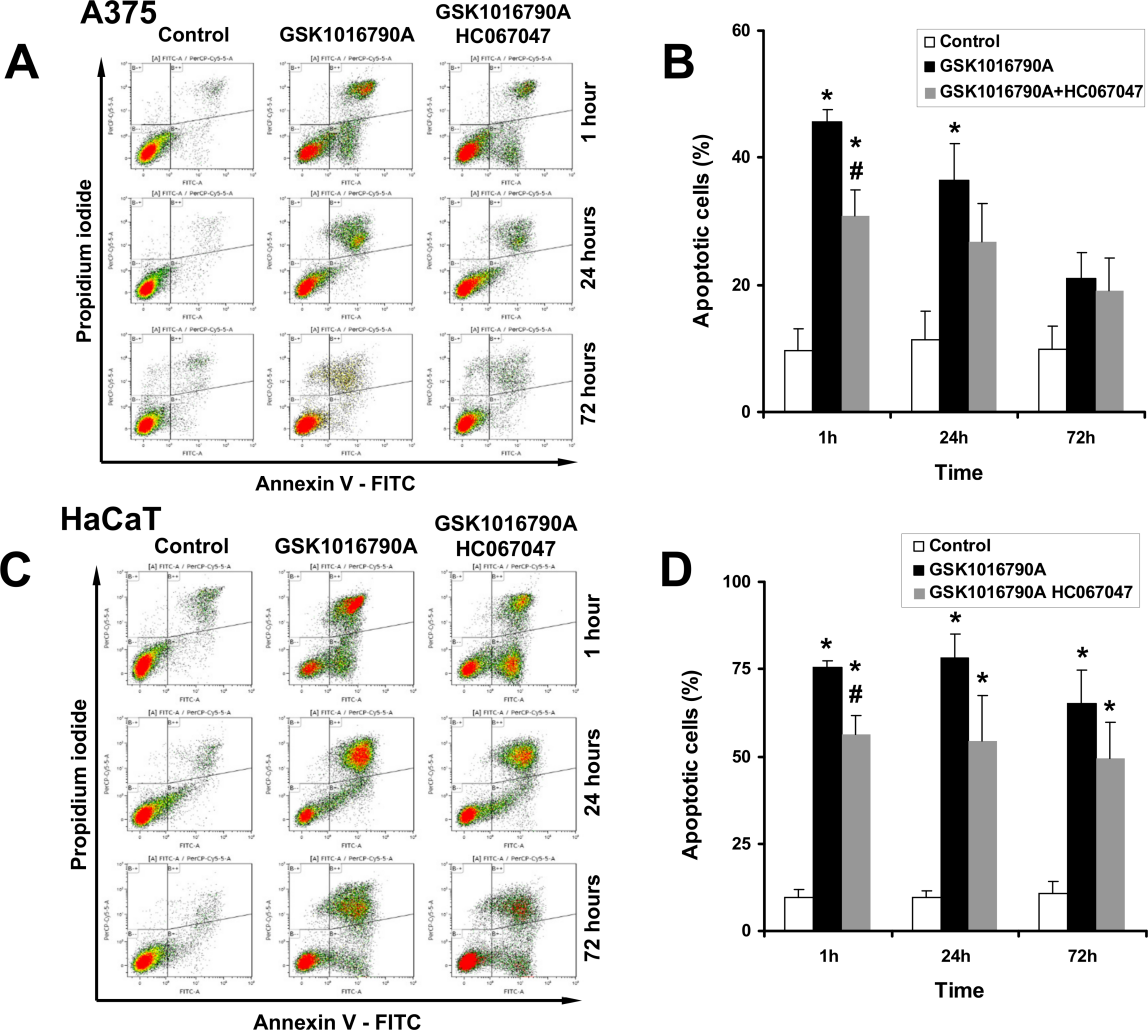
FACS analysis of apoptosis. A) Representative flow cytometry dot plots with double Annexin V-FITC/PI staining for control cells (DMSO 0,2%), cells exposed to GSK1016790A (10 nM), and cells exposed to GSK1016790A and HC067047 (1 μM) at 1 h, 24 h, and 72 h. B) Summary data. C) Induction of apoptosis in HaCaT cells and protective effects of HC067047. D) Summary data. *P<0.05 vs. Control, #P<0.05 vs. GSK1016790A, ANOVA, n = 3). Data are means ± SEM (number of independent experiments, n = 3).

To gain further insights into the cytotoxicity of pharmacological TRPV4 activation, we measured induction of apoptosis over 3 days in the continuing presence of GSK1016790A (Fig 5).

TRPV4 activation increased the percentage of apoptotic cells (Annexin V+) to 45% of all cells after 1 h, which was significantly different from DMSO-controls (10%; Fig 5A for representative plots and Fig 5B for summary data). Of the GSK1016790A-treated apoptotic cells, 15% was in an early stage of apoptosis (Annexin V+/PI-) and the rest was in a late apoptotic stage, or secondary necrosis (Annexin V+/PI+). When HC067047 was co-administered, percentages of all, early, and late apoptotic cells were significantly less (30%). Thus, HC067047 counteracted GSK1016790A-induced apoptosis but did not fully prevent it, which could be explained by the slow kinetics of channel blockade as mentioned above.

The pro-apoptotic effect of GSK1016790A was still observed after 24 h- and 72 h- exposure, although the percentage of all apoptotic cells (Annexin V+) progressively decreased, in particular the percentage of early apoptotic cells, while the percentage of necrotic cells (Annexin V-/PI+) increased.

Regarding HaCaT cells, we found a similar induction of apoptosis in response to GSK1016790A (Fig 5C and 5D). Inhibition of TRPV4 by HC067047 prevented these responses to GSK1016790A in a significant manner and to a similar degree as in A375 cells.

These data show that pharmacological TRPV4 activation produced apoptosis in A375 cells and HaCaT keratinocytes.

### Impact of TRPV4 activation on the cell cycle

**Fig 6:**
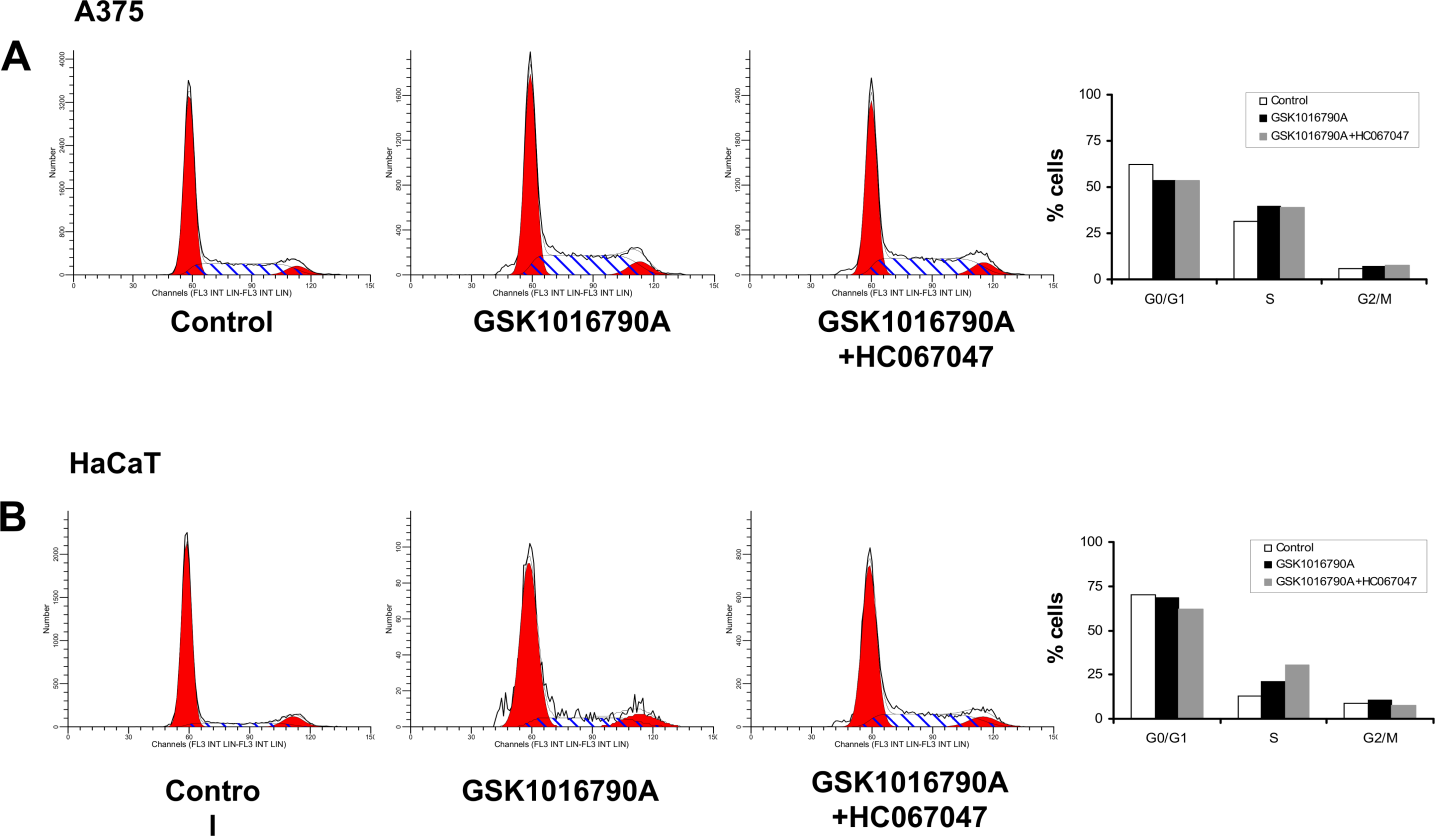
Pharmacological TRPV4 activation did not impair the cell cycle in A375 cells (A) and HaCaT keratinocytes (B).

We also studied whether GSK1016790A affected progression of A375 cells and HaCaT keratinocytes through the cell cycle (Fig 6). Here, the FACS analysis revealed that the overall lower number of A375 cells surviving the first 24h of TRPV4 activation had a slightly lower percentage of cells in GO/G1 and a slightly higher percentage of cells in S phase but no change in G2/M if compared to controls (Fig 6A). Moreover, a noteworthy portion of cells was found in sub-G1 (necrotic/late apoptotic cells), which is in line with the higher rate of apoptosis mentioned above. Such cells were not found in DMSO-treated controls. HC067047 did not prevent the moderate alterations of percentages of cells being in G0/G1 or the S-phase but lowered the percentage of cells in sub-G1, which again is in line with the protective effects of this TRPV4 blocker previously seen in the apoptosis assays.

In HaCaT keratinocytes we found the same alterations of the cell cycle in response to GSK1016790A and the combination of GSK1016790A and HC067047 (Fig 6B).

Together, these data suggested that pharmacological TRPV4 activation did not interfere with cell cycle progression of surviving cells in an appreciable fashion.

### Impact of TRPV4 activation on cell proliferation and survival

**Fig 7:**
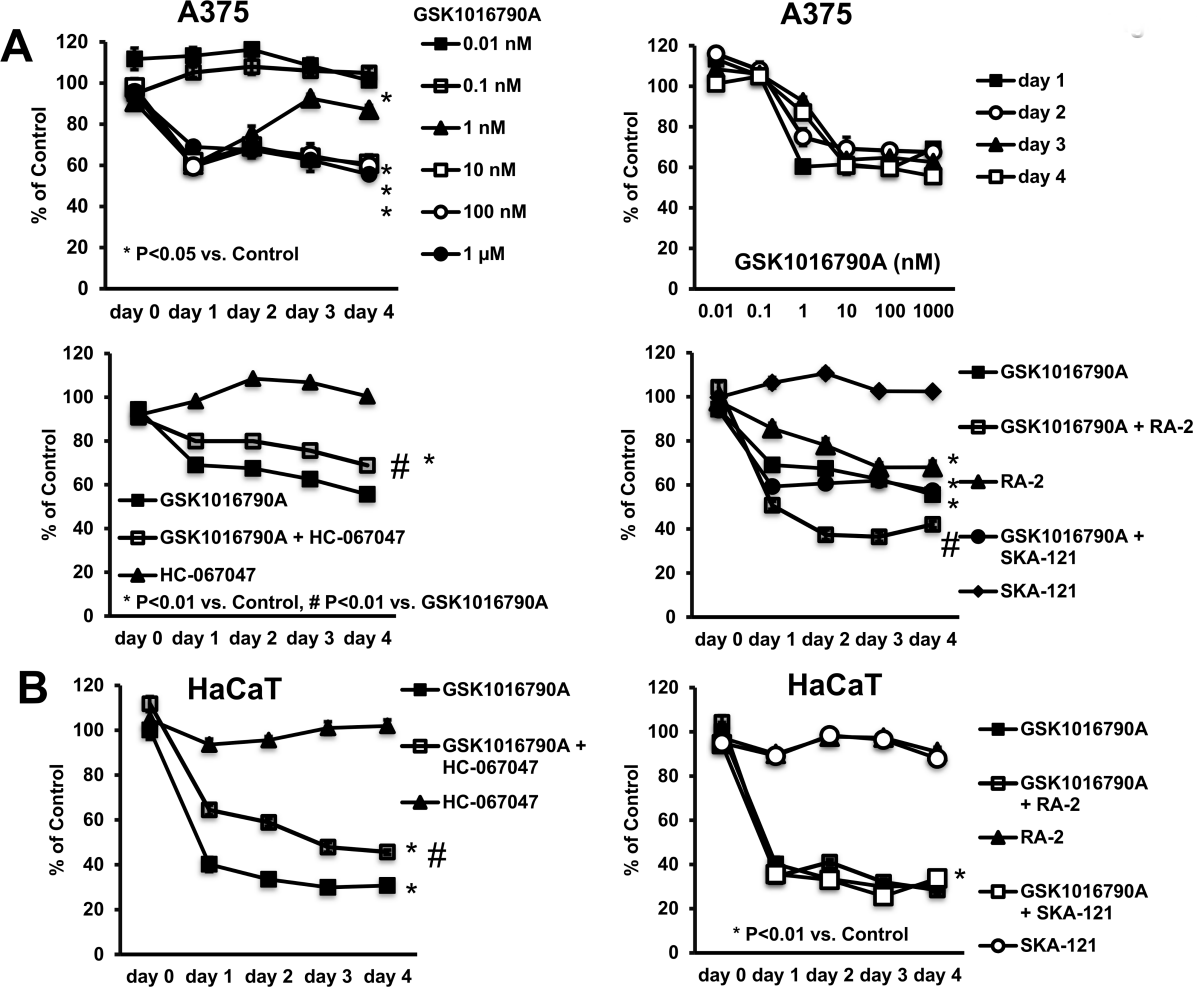
Impact of GSK1016790A on cell proliferation/survival. A) Upper panel on left: Concentration-dependent reduction of cell proliferation/survival of A375 as measured by Janus Green-Assay. Upper panel on right: Note that half-maximal inhibition was achieved at ca. 1 nM GSK1016790A for all time intervals, except day-1. Lower panel on left: HC067047 antagonized the response to GSK1016790A. Lower panel on right: The negative gating-modulator of KCa3.1, the 13b derivate, RA-2 (10 μM) reduced cell proliferation/survival and potentiated the response to GSK1016790A. The positive-gating modulator of KCa3.1, SKA-121, had no effects. Data points are means ± SEM (n = 6–36 from n = 2–6 independent experiments). B) GSK1016790A impaired proliferation/survival of HaCaT cells. HC067047 partially antagonized the response. The negative and positive KCa3.1-gating modulators, RA-2 and SKA-121, respectively, did not modulate the response. Data points are means ± SEM (n = 18; number of independent experiments, n = 3). *P<0.05 vs. DMSO, #P<0.05 vs. GSK1016790A; Student’s T test.

Lastly, we wanted to examine the effect of TRPV4 activation on cell proliferation and survival of A375 cells and HaCaT keratinocytes. To do this we used the Janus Green assay, a method for measuring cell proliferation/survival over 5-days. These experiments revealed that TRPV4 activation by GSK1016790A inhibited cell proliferation/survival of A375 cells in a concentration-dependent fashion. A half maximal effect was obtained at ≈1 nM (Fig 7A, upper panels), which corresponded well to the previously reported EC_50_ for channel activation [1,13]. This anti-proliferative effect was antagonized, though again not completely by inhibition of TRPV4 (Fig 7A, lower panel on left).

In keeping with the presence of calcium/calmodulin-regulated KCa3.1 channels in A375 cells (Fig 1) and their co-activation in response to GSK1016790A, we also tested whether an inhibitor of KCa3.1, the 13b-derivate, RA-2 [21], and the KCa3.1-selective activator, SKA-121, modulated GSK1016790A-induced inhibition of cell proliferation/survival and had effects on their own (Fig 7A, lower panel on right). RA-2 significantly augmented the impact of GSK1016790A, and mildly reduced A375 proliferation/survival on its own. The KCa3.1-activator, SKA-121, did not modulate the impact of GSK1016790A and had no impact on its own. So, these data suggested that the effect of TRPV4-activation and KCa3.1-inhibtion were additive.

HaCaT cell proliferation/survival was similarly impaired by GSK1016790A (Fig 7B) and HC067047 prevented this. The positive and negative KCa3.1-gating modulators had also no significant impact, either alone or in combination with GSK1016790A. To the contrary, we did not see an impact of GSK1016790A on 3T3 fibroblast proliferation (S2 Fig).

## Discussion

The present in-vitro study characterized TRPV4 channels in human melanoma cell lines and keratinocytes and suggests a utility of small molecule TRPV4 activators to impede cell proliferation and survival. This conclusion is based on following observations: 1. We demonstrated TRPV4 gene expression and TRPV4 channel membrane function in melanoma cell lines and non-cancer HaCaT keratinocytes. 2) Pharmacological activation of TRPV4 and ensuing calcium-influx caused immediate cellular disarrangement and cell death in all lines. 3) Pharmacological channel activation promoted apoptosis and strongly inhibited cell proliferation/survival.

TRPV4 channel function has previously been described in a variety of tissues such as endothelium, cell layers of circumferential organs, renal epithelium, and skin to name some [1,2,3,7,8,28,29]. Information about TRPV4 in cancer tissues is scarce. Here, our electrophysiological study provides new knowledge by showing functional expression of TRPV4 channels in the cell membrane of A375 melanoma cells as undoubtedly concluded from the pharmacological and biophysical fingerprints of the current (Figs 1 and 2), such as, activation by the selective activator, GSK1016790A, inhibition of currents by selective inhibitor, HC067047, mild N-shape IV relationship, and calcium permeability. A similar functional expression and gene expression of TRPV4 was found in the amelanotic melanoma cell line, A375, and the melanotic melanoma lines MKTBR and SK-MEL-28, which is important to know since melanogenesis modulates expression of several genes such as the hypoxia-inducible factor-1 and related pathways and may thus decide on the availability of a specific target or targeted pathway and/or cancer progression [30].

Similar to reports on TRPV4 in other cell types [31,32], TRPV4 synergistically interacted with calcium/calmodulin-gated KCa3.1 because the latter channel was co-activated as consequence of TRPV4-mediated calcium-entry (Fig 1). At the molecular level, the finding of TRPV4-mRNA and of KCa3.1, further supported functional expression of these channels in A375 cells.

Our calcium imaging experiments (Fig 3) further complemented the experimental evidence by demonstrating increases of [Ca^2+^]_i_ following TRPV4 activation and substantial and probably cytotoxic calcium-overload that could only be partially reversed by TRPV4 blockade.

Non-cancer HaCaT cells also expressed functional TRPV4 channels as proven by patch-clamp and qRT-PCR. Our qRT-PCR also detected KCa3.1 mRNA-expression but we failed to record KCa3.1 currents following TRPV4 activation, presumably because of very low membrane expression of functional channels or few KCa3.1-expressing cells. When compared with A375 cells, mRNA-expression levels for both channels in HaCaT keratinocytes were 10-times lower. Regarding [Ca^2+^]_i_, we likewise demonstrated a strong increase of [Ca^2+^]_i_ in response to GSK1016790A. TRPV4 expression in 3T3 fibroblasts was considerably lower than in the other cell types, but currents resembled biophysical and pharmacological properties of TRPV4 [1,2,3,4,5,6,7].

A major goal of the present study was to determine whether pharmacological manipulation of TRPV4 has any consequences for melanoma cell survival and/or proliferation. Here our study provides new insight as we could show that pharmacological activation of TRPV4 considerably impaired melanoma cell survival and proliferation (Fig 7). Moreover, the effects of channel activation could be prevented to a large extent by the TRPV4-blocker, HC067047, suggesting that the effects can indeed be attributed to TRPV4-activation. It is noteworthy that inhibition of TRPV4 had no effect in the present study suggesting that normal physiological functions of the channel are not required here.

The deleterious effect of TRPV4 channel activation appeared to rely on two temporally distinct mechanisms: an instantaneous mechanism characterized by immediate cellular disarrangement, pyknosis, retraction of lamellipodia, blebbing, cell rounding and detachment (Fig 4), resembling typical features of necrosis. These responses were most likely a consequence of the strong calcium (and sodium) entry through TRPV4 and, presumably, the concomitant activation of a broad range of intracellular calcium-dependent pathways including cytoskeletal re-arrangements and breakdown of ionic gradients. The other mechanism seemed to be the induction of apoptosis—likewise calcium-dependent—that occurred over longer time interval (hours to days, Fig 5).

A clear effect on cell cycle progression was, however, not evident.

In summary, it seems to be clear that TRPV4 activation in A375 cells produced substantial cytotoxicity and it is therefore worth speculating that the cytotoxicity of this or other TRPV4 activators could help to impede melanoma progression and metastasis. However, this needs to be proven experimentally in future studies.

Considering TRPV4 a thermosensitive channel [5], it might be worth testing the impact of TRPV4 activation in combination with radiotherapy to enhance the latter’s efficacy, particularly in pigmented melanoma lines. It is also worth testing this together with inhibitors of melanogenesis that have been demonstrated to enhance efficacy of radiotherapy in pigmented melanoma [19,33,34].

The apparent and immediate cytotoxicity of TRPV4-activation was not limited to A375 since the other melanoma lines, MKTBR and SK-MEL-28, responded similarly to TRPV4-activaton within the first hour (S1 Fig). Also, the non-cancer keratinocyte line, HaCaT, was found to be sensitive to TRPV4 activation (S1 Fig) since we observed instantaneous membrane blebbing and pyknosis, cell detachment, and necrosis. Again, similar to A375 cells, we found significant induction of apoptosis.

These findings in HaCaT keratinocytes provided additional evidence that TRPV4 is an epidermal channel that may not only mechanistically linked to barrier functions [2,8], but according to the present study also to cell proliferation. From the dermatologist’s perspective, pharmacological activation of the keratinocyte channel could be a way to halt hyperkeratosis in autoimmune disease, such as psoriasis. Interestingly, a recent study in rosacea patients suggested pathological relevant roles of TRPV4 in this skin disease, although activation of TRPV4 in mast cells was shown to be the main driver here [35,36]. Another study reported that the flavonoid, baicalein, increased keratin 1 and 10 production by using TRPV4 channels, which further supports TRPV4 as potential drug target in skin disease [37].

In keeping with the frequent and apparently calcium-influx dependent co-activation of KCa3.1 in A375 cells, we found that inhibition of this channel had also anti-proliferative effects, but they were less pronounced. Still, this is in line with the idea that KCa3.1 has an oncogenic potential in melanoma, in this regard similar to earlier reports on pro-proliferative and/or pro-migratory roles and cancer progression promoting role in several other solid cancers, like breast, lung, kidney, and pancreatic cancer [23,38,39,40], as well as in chronic lymphocytic leukemia [41]. It is noteworthy that also a close relative of KCa3.1, KCa2.3, has been suggested to play roles, particularly in melanoma motility [42]. In contrast to KCa3.1 inhibition, activation of KCa3.1 with SKA-121 did not modulate melanoma A375 proliferation (Fig 7), suggesting the amplified channel functions do not play major roles here.

Considering the functional interplay of KCa3.1 and TRPV4 reported here, the anti-proliferative actions of pharmacological inhibition of KCa3.1 and activation of TRPV4 had additive effects but no apparent supra-additive, potentiating effects. In contrast, pharmacological activation of KCa3.1 did not alter the anti-proliferative actions of TRPV4 activation. This suggested that KCa3.1 co-activation is not a requirement for the alterations caused by pharmacological TRPV4-activation, but KCa3.1’s normal function may promote on its own A375 mitogenesis, thus supporting its oncogenic potential and the channel as target for adjuvant treatments [43,44].

In conclusion, our study described expression and function of TRPV4 (and KCa3.1) channels in melanoma cells and revealed a potential utility of TRPV4-activators as alternative or adjuvant therapeutic strategy to produce melanoma cell death and to impede tumor progression.

## Supporting information

S1 Fig TRPV4 and KCa3.1 gene expression. Comparative quantitative RT-PCR analysis of channel expression as percentage of GAPDH expression in A375, SK-MEL-28, and MKTBR (replicates, n = 3). Data points are means ± SEM.
(PDF)

S2 Fig Cell morphology. A) Exemplary light microscopic images of GSK1016790A-induced morphological alterations in MKTBR melanoma cells after 1 h and protective effect of HC067047. B) Time course of morphological alterations of HaCaT keratinocytes during the first hour of exposure to GSK1016790A (1 μM). HC067047 (1 μM) prevented GSK1016790A-induced alterations. HC067047 or vehicle (DMSO) had no apparent effect. Lower left panels: Giemsa-stained HaCaT cells after 1 h exposure to GSK1016790A, in combination with HC067047, or DMSO. On right: Counts of non-viable, “death” cells in supernatant. Data points are means ± SEM (number of independent experiments, n = 3).
(PDF)

S3 Fig Electrophysiological properties of TRPV4 in murine 3T3 fibroblasts. A) Mean TRPV4-currents activated by GSK1016790A (200 nM) and inhibition by HC067047. Note that current amplitudes were smaller than in melanoma cell lines and HaCaT cells. Data points are means ± SEM (n = 5). B) Exemplary whole-cell recording (1 μM). C) Light microscopic images of 3T3 fibroblast treated with DMSO (vehicle), GSK1016790A (1 μM) alone or in combination with HC067047 (1 μM), and with HC067047. Note that cells remained morphologically intact. D) GSK1016790A at 10 μM did not modulate cell proliferation/survival (n = 4).
(PDF)

S1 Appendix Data TRPV4 currents in SKMEL-28. (XLS)

S2 Appendix Data TRPV4 currents in 3T3. (XLS)

S3 Appendix Data TRPV4 currents in MKTBR. (XLS)

S4 Appendix Data TRPV4 currents in A375. (XLS)

S5 Appendix Data TRPV4 currents in HaCaT. (XLS)

S6 Appendix Data qRTPCR HACAT A375 MKTBR SKMEL-28. (XLS)

S7 Appendix Data optical mapping. (XLS)

S8 Appendix Apoptosis. (XLS)

S9 Appendix Cell cycle analysis. (XLS)

S10 Appendix Dead cells in suspension. (XLS)

S11 Appendix Data Janus Green Assay. (XLS)

## Abbreviations

TRPV4: transient receptor potential vanilloid-type 4 channel
KCa3.1: intermediate-conductance calcium-activated K channel
