# Supplementary material for: Pharmacological activation of TRPV4 produces immediate cell damage and induction of apoptosis in human melanoma cells and HaCaT keratinocytes
Source: PLoS One. 2018 Jan 2;13(1):e0190307. doi: 10.1371/journal.pone.0190307 (PMC5749757; doi:10.1371/journal.pone.0190307)
Supplement: S1 Fig — Comparative quantitative RT-PCR analysis of channel expression as percentage of GAPDH expression in A375, SK-MEL-28, and MKTBR (replicates, n = 3). Data points are means ± SEM. (PDF) [file pone.0190307.s001.pdf]

Suppl. Figure 1

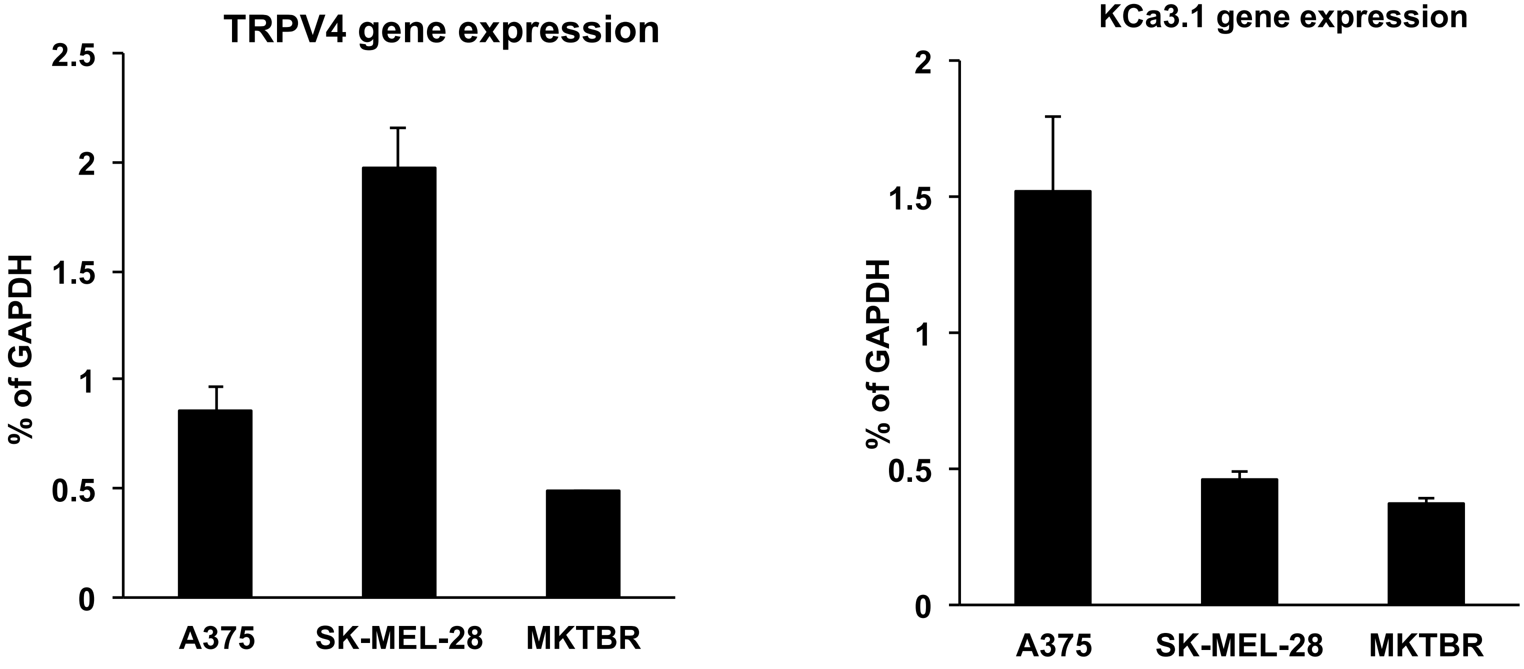

S1 Fig. TRPV4 and KCa3.1 gene expression. Comparative quantitative RT-PCR analysis of channel expression as percentage of GAPDH expression in A375, SK-MEL-28, and MKTBR (replicates, n=3). Data points are means ± SEM.
