# Supplementary material for: Pharmacological activation of TRPV4 produces immediate cell damage and induction of apoptosis in human melanoma cells and HaCaT keratinocytes
Source: PLoS One. 2018 Jan 2;13(1):e0190307. doi: 10.1371/journal.pone.0190307 (PMC5749757; doi:10.1371/journal.pone.0190307)
Supplement: S2 Fig — A) Exemplary light microscopic images of GSK1016790A-induced morphological alterations in MKTBR melanoma cells after 1 h and protective effect of HC067047. B) Time course of morphological alterations of HaCaT keratinocytes during the first hour of exposure to GSK1016790A (1 μM). HC067047 (1 μM) prevented GSK1016790A-induced alterations. HC067047 or vehicle (DMSO) had no apparent effect. Lower left panels: Giemsa-stained HaCaT cells after 1 h exposure to GSK1016790A, in combination with HC067047, or DMSO. On right: Counts of non-viable, “death” cells in supernatant. Data points are means ± SEM (number of independent experiments, n = 3). (PDF) [file pone.0190307.s002.pdf]

Suppl. Figure 2

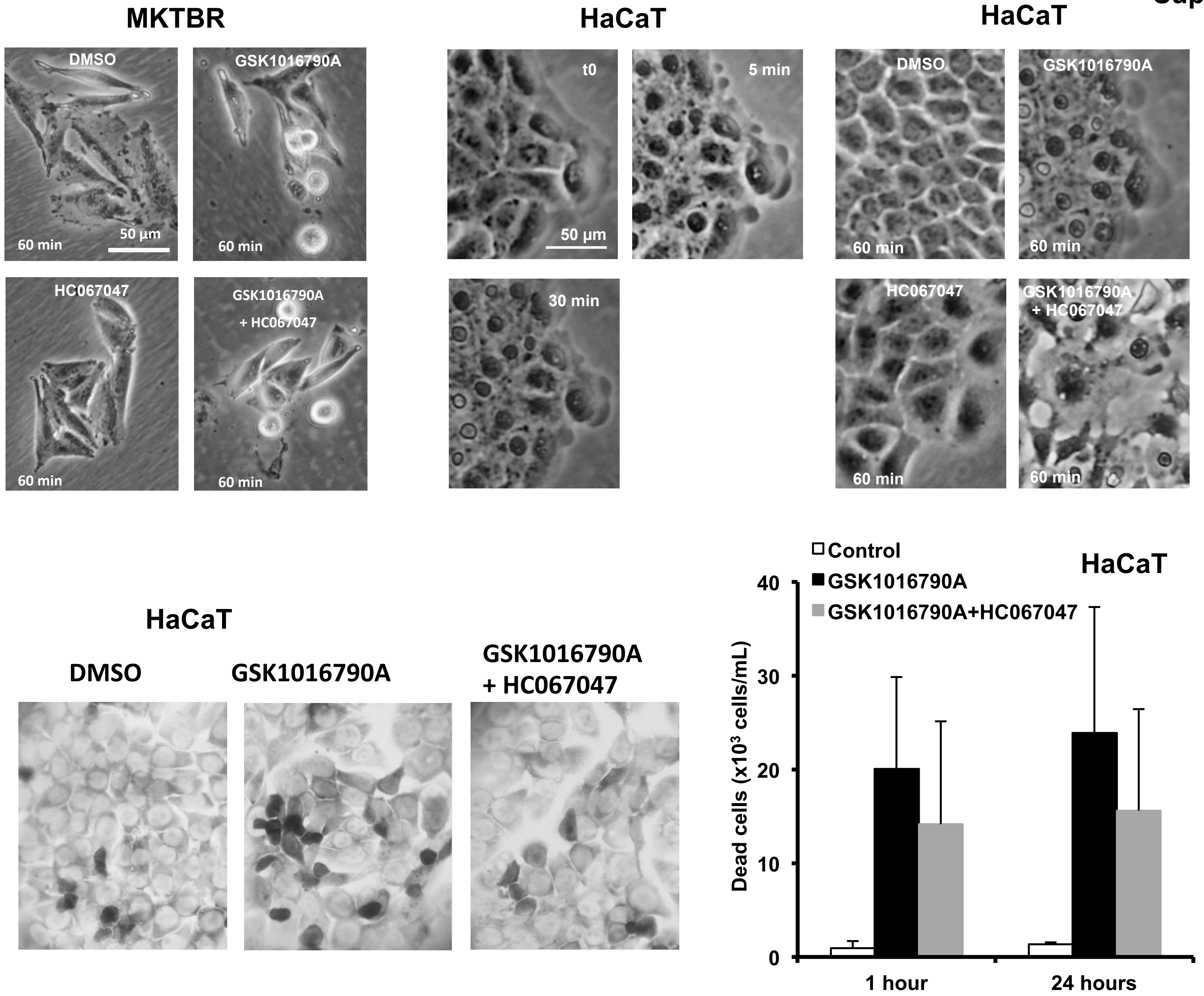

S2 Fig. Cell morphology. A) Exemplary light microscopic images of GSK1016790A-induced morphological alterations in MKTBR melanoma cells after 1 h and protective effect of HC067047. B) Time course of morphological alterations of HaCaT keratinocytes during the first hour of exposure to GSK1016790A (1 µM). HC067047 (1 µM) prevented GSK1016790A-induced alterations. HC067047 or vehicle (DMSO) had no apparent effect. Lower left panels: Giemsa-stained HaCaT cells after 1 h exposure to GSK1016790A, in combination with HC067047, or DMSO. On right: Counts of non-viable, "death" cells in supernatant. Data points are means ± SEM (number of independent experiments, n=3).
