# Supplementary material for: Pharmacological activation of TRPV4 produces immediate cell damage and induction of apoptosis in human melanoma cells and HaCaT keratinocytes
Source: PLoS One. 2018 Jan 2;13(1):e0190307. doi: 10.1371/journal.pone.0190307 (PMC5749757; doi:10.1371/journal.pone.0190307)
Supplement: S3 Fig — A) Mean TRPV4-currents activated by GSK1016790A (200 nM) and inhibition by HC067047. Note that current amplitudes were smaller than in melanoma cell lines and HaCaT cells. Data points are means ± SEM (n = 5). B) Exemplary whole-cell recording (1 μM). C) Light microscopic images of 3T3 fibroblast treated with DMSO (vehicle), GSK1016790A (1 μM) alone or in combination with HC067047 (1 μM), and with HC067047. Note that cells remained morphologically intact. D) GSK1016790A at 10 μM did not modulate cell proliferation/survival (n = 4). (PDF) [file pone.0190307.s003.pdf]

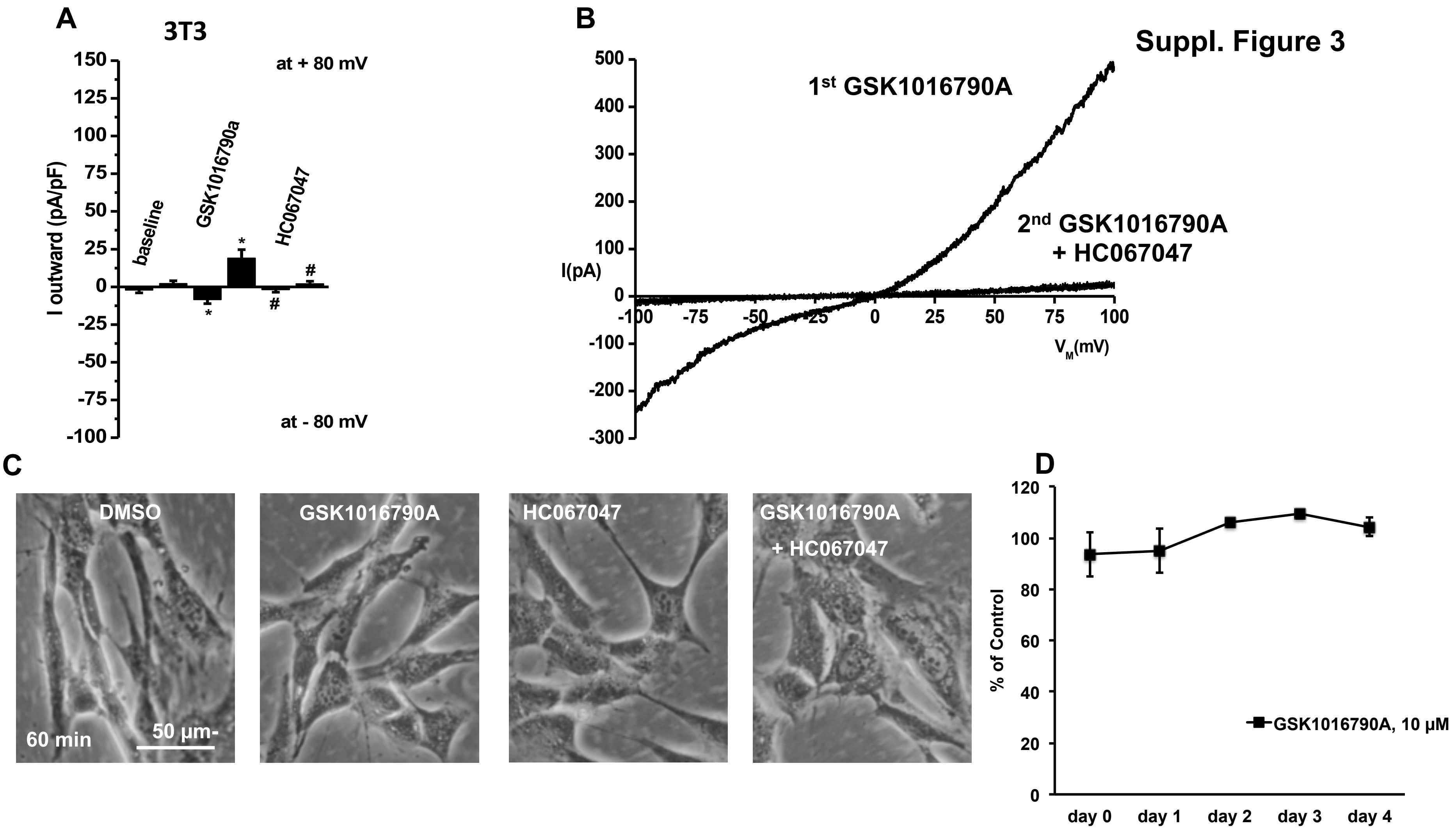

S3 Fig. Electrophysiological properties of TRPV4 in murine 3T3 fibroblasts. A) Mean TRPV4-currents activated by GSK1016790A (200 nM) and inhibition by HC067047. Note that current amplitudes were smaller than in melanoma cell lines and HaCaT cells. Data points are means ± SEM (n=5). B) Exemplary whole-cell recording (1 µM). C) Light microscopic images of 3T3 fibroblast treated with DMSO (vehicle), GSK1016790A (1 µM) alone or in combination with HC067047 (1 µM), and with HC067047. Note that cells remained morphologically intact. D) GSK1016790A at 10 µM did not modulate cell proliferation/survival (n=4).
